# O’nyong’nyong Virus Infections in Nigeria: A Case Series

**DOI:** 10.1093/cid/ciaf511

**Published:** 2025-11-20

**Authors:** Cyril Erameh, Vivian Kwaghe, Ephraim Ogbaini-Emovon, Jay Osi Samuels, Osahogie Isaac Edeawe, Nankpah Godsave Vongdip, Onyia Justus Ejike, Blessed Okhiria, Ikponmwosa Odia, Jacqueline Agbukor, Lauren P Courtney, Adamu Zigwai Ephraim, Claire A Quiner, Jean H Kim, Philippe Chebu, Oladimeji Damilare Matthew, Victoria Orok, Walter Mary Odion, Blessing Amierhobhiye Obagho, Richard Fayomade, Reuben Eifediyi, Emmanuel A Oga

**Affiliations:** Institute of Viral and Emergent Pathogens Control and Research, Irrua Specialist Teaching Hospital, Edo, Nigeria; Internal Medicine, University of Abuja Teaching Hospital, Gwagwalada, Federal Capital Territory, Nigeria; Institute of Viral and Emergent Pathogens Control and Research, Irrua Specialist Teaching Hospital, Edo, Nigeria; Laboratory Services, APIN Public Health Initiatives, Federal Capital Territory, Nigeria; Institute of Viral and Emergent Pathogens Control and Research, Irrua Specialist Teaching Hospital, Edo, Nigeria; Internal Medicine, University of Abuja Teaching Hospital, Gwagwalada, Federal Capital Territory, Nigeria; Internal Medicine, University of Abuja Teaching Hospital, Gwagwalada, Federal Capital Territory, Nigeria; Internal Medicine, University of Abuja Teaching Hospital, Gwagwalada, Federal Capital Territory, Nigeria; Institute of Viral and Emergent Pathogens Control and Research, Irrua Specialist Teaching Hospital, Edo, Nigeria; Institute of Viral and Emergent Pathogens Control and Research, Irrua Specialist Teaching Hospital, Edo, Nigeria; Solutions, RTI International, Durham, North Carolina, USA; Solutions, RTI International, Durham, North Carolina, USA; Solutions, RTI International, Durham, North Carolina, USA; Solutions, RTI International, Durham, North Carolina, USA; Laboratory Services, APIN Public Health Initiatives, Federal Capital Territory, Nigeria; Internal Medicine, University of Abuja Teaching Hospital, Gwagwalada, Federal Capital Territory, Nigeria; Internal Medicine, University of Abuja Teaching Hospital, Gwagwalada, Federal Capital Territory, Nigeria; Institute of Viral and Emergent Pathogens Control and Research, Irrua Specialist Teaching Hospital, Edo, Nigeria; Institute of Viral and Emergent Pathogens Control and Research, Irrua Specialist Teaching Hospital, Edo, Nigeria; Laboratory Services, APIN Public Health Initiatives, Federal Capital Territory, Nigeria; Institute of Viral and Emergent Pathogens Control and Research, Irrua Specialist Teaching Hospital, Edo, Nigeria; Solutions, RTI International, Durham, North Carolina, USA; ClineEpi Partners, Columbia, Maryland, USA

**Keywords:** o’nyong’nyong virus, arbovirus, febrile illness, Nigeria, TaqMan Array Card

## Abstract

We report seven patients with detected o’nyong’nong virus infection, presenting with acute febrile illness at two tertiary hospitals in Nigeria. The clinical presentation mimicked malaria and Lassa fever, highlighting the need for expanded arboviral surveillance and diagnostics to distinguish emerging viral infections in endemic settings.

O’nyong’nyong virus (ONNV) is a mosquito-borne alphavirus within the Togaviridae family, first isolated in Uganda in 1959 during a large outbreak of polyarthritis and fever [1]. It is transmitted primarily by *Anopheles gambiae* and *Anopheles funestus*, and its clinical presentation often mimics other endemic tropical diseases such as malaria and Lassa fever [2]. Although widespread serological evidence of ONNV exists across Central and West Africa, confirmed clinical cases remain rare, partly due to limited surveillance and diagnostic capabilities [3]. ONNV presents with fever, headache, and joint pains, particularly the large joints. There may be a rash, maculopapular and itchy. Posterior cervical lymphadenopathy may be seen on examination. To the best of our knowledge, no known reports of ONNV have been detected in Nigeria.

This report presents the first detection of ONNV infection in a clinical setting from two hospitals in north-central and southern Nigeria: University of Abuja Teaching Hospital (UATH) in the Federal Capital Territory (FCT) and Irrua Specialist Teaching Hospital (ISTH) in Edo State. These patients were detected between September 2023 to May 2024 as part of the Surveillance of Acute Febrile Illness Aetiology in Nigeria (SAFIAN) study which enrolled 1200 participants [4]. ONNV pathogen detection was performed using Thermo Fisher's TaqMan Array Card (TAC) platform, which utilizes reverse-transcription polymerase chain reaction (RT-PCR) technology as a screening tool. TAC is designated for research use only (RUO) and is not intended for clinical diagnosis. Details on the TAC panel configuration, assay performance, and broader pathogen detection and outcomes from the SAFIAN study are provided in the primary outcomes paper [3]. Lassa virus PCR testing was conducted on all samples using the RealStar Lassa Virus RT-PCR kit (Altona Diagnostics; Hamburg, Germany), following hospital and study protocols. Malaria testing was conducted by the hospitals according to their standard clinical protocols.

## CASE PRESENTATIONS

### Case 1

A 10-year-old female student from Gwagwalada, FCT, presented to the UATH general outpatient department with a 4-day history of headaches and runny nose. Her temperature was 38.3°C. She had below primary-level education and no known underlying conditions. Hospital microscopy was positive for malaria, although TAC-PCR testing for *Plasmodium* spp. was negative, ONNV was detected by TAC. She received antimalarial treatment and was discharged to home the same day.

### Case 2

A 16-year-old male student from Bwari, FCT, presented to the emergency pediatric unit of UATH with an 8-day history of diarrhea and fever. His axillary temperature was 37.9°C. He had no known prior health conditions. TAC for arboviruses returned positive for ONNV. Malaria was detected via hospital microscopy, but testing for *Plasmodium* spp. via TAC returned negative. Patient was treated with antimalarials and discharged after one day.

### Case 3

A 76-year-old retired nurse from Lokoja, Kogi State, presented to the UATH medical emergency unit with a 2-day history of fever and cough. Her recorded temperature was 39.4°C. Patient was treated with intravenous (IV) paracetamol (PCM) and passed away after one day. TAC testing for arboviruses returned positive for ONNV only. She had no documented coinfections.

### Case 4

A 25-year-old female civil servant from Esan Central, Edo State, presented to the ISTH general outpatient clinic with a one-day history of fever, generalized body pain, malaise, and abdominal discomfort. Her axillary temperature was 39°C. She had no history of recent travel or livestock contact and reported no insect bites. Malaria was detected via hospital microscopy but testing for *Plasmodium* via TAC was negative. TAC testing for arboviruses returned positive for ONNV. She was treated with oral artemether-lumefantrine (Lokmal) and ibuprofen. Her symptoms resolved within days without any complications.

### Case 5

A 25-year-old unemployed female from Esan West, Edo State, presented to the ISTH general outpatient clinic with a two-day history of fever, abdominal pain, diarrhea, and headache. Her tympanic temperature at the hospital was 37.4°C. She had a history of dyspepsia and reported frequent mosquito bites. The malaria rapid diagnostic test (RDT) was positive, although PCR testing for *Plasmodium* spp. was negative using TAC. Additionally, confirmatory Lassa fever virus testing via RealStar Lassa Virus RT-PCR kit was negative. The TAC-PCR test detected ONNV. Treatment included Lokmal, paracetamol, oral Augmentin, and omeprazole. She was hospitalized for two days. She recovered fully and was discharged without sequelae.

### Case 6

A 33-year-old unemployed female from Etsako West, Edo State, presented to the ISTH accident and emergency department with a 4-day history of cough and abdominal pain. Her temperature was 37.8°C. TAC-PCR detected *Plasmodium* spp., and Lassa fever PCR was positive. TAC for arboviruses returned as positive detection for ONNV. Patient was treated with Lokmal and Augmentin. She was hospitalized for six days and discharged in good condition.

### Case 7

A 38-year-old housewife from Esan West, Edo State, presented to the ISTH accident and emergency department with a one-day history of headache. Her temperature was 37.6°C. The malaria RDT was positive, but testing for *Plasmodium* via TAC returned negative. TAC detected ONNV and a coinfection of Rickettsiosis. She was treated with antimalarials and discharged after one day without complications.

A summary of clinical and demographic characteristics is shown in Table 1, including hospital diagnosis and treatment.

**Table 1.** Clinical and Demographic Characteristics of Patients Testing Positive for ONNV at Two Tertiary Hospitals in Edo State and FCT, Nigeria

Characteristic	Case 1	Case 2	Case 3	Case 4	Case 5	Case 6	Case 7
Site	UATH	UATH	UATH	ISTH	ISTH	ISTH	ISTH
Hospital department	General outpatient department	Emergency pediatric unit	Medical emergency unit	General outpatient clinic	General outpatient clinic	Accident and emergency	Accident and emergency
Age	10	16	76	25	25	33	38
Sex	Female	Male	Female	Female	Female	Female	Female
Month of enrollment	December	November	February	September	November	January	May
Coinfections detected through TAC RT-PCR	…	…	…	…	…	Malaria (TAC-PCR)	Rickettsiosis (TAC-PCR)
Hospital-detected Coinfections	Malaria (microscopy)	Malaria (microscopy)	…	Malaria (microscopy)	Malaria (RDT)	Lassa fever Positive (PCR)	Malaria (RDT)
Other health conditions	None	None	None	None	PUD	None	None
Symptoms	Runny nose, headache	Diarrhea	Cough	Abdominal pain	Diarrhea, abdominal pain, headache	Cough, abdominal pain	Headache
Temperature at initial hospital intake	38.3°C	37.9°C	39.4°C	39°C	37.4°C (self-reported fever)	37.8°C	37.6°C
Number of days with fever prior to hospitalization	4	8	2	4	2	4	1
If admitted, number of days in hospital	1/2	1	1	Not admitted	2	6	1
Patient self-treatment	Antimalaria, PCM	Don’t know	Antibiotics	Coartem, ibuprofen	Lokmal omeprazole, PCM	Lokmal, PCM	PCM
Final hospital diagnosis	Malaria	Malaria	Unknown	Malaria	Malaria	Lassa fever	Malaria
Hospital treatment	Antimalaria	Antimalaria	IV PCM, CVD, HTN	Lokmal	Lokmal, Augmentin	Ribavirin, ceftriaxone	Antimalarial
Disposition at discharge	Discharged to home	Discharged to home	Deceased	Discharged to home	Discharged to home	Discharged to home	Discharged to home

Abbreviations: CVD, cardiovascular disease; HTN, hypertension; ISTH, Irrua Specialist Teaching Hospital; ONNV, o’nyong’nyong virus; PCM, paracetamol; PUD, peptic ulcer disease; RDT, rapid diagnostic test; TAC-PCR, TaqMan Array Card polymerase chain reaction; UATH, University of Abuja Teaching Hospital.

## DISCUSSION

The overlap in symptoms between o’nyong’nyong fever and other endemic febrile illnesses such as malaria or Lassa fever often leads to diagnostic confusion, especially in the absence of molecular diagnostics. Notably, two of our cases tested positive on malaria RDT and three tested positive via microscopy, but these results were contradicted by the TAC. Use of TAC requires confirmatory testing, since this is a research-use only screening instrument. However, these findings suggest a limitation of presumptive diagnosis and the potential for false-positive microscopy or RDTs [5].

The detection of ONNV in this case series highlights the potential of TAC as a powerful tool for pathogen identification. However, it is important to note that this platform is currently designated for research use only and has not been validated for routine clinical diagnostics, necessitating confirmatory testing with clinically approved assays. Despite this limitation, multiplex platforms such as TAC offer significant value by enabling simultaneous screening for a broad spectrum of viral, bacterial, and parasitic pathogens—supporting syndromic, algorithm-based approaches to diagnosing febrile illnesses, particularly in resource-limited, high-burden settings.

The identification of ONNV in patients initially diagnosed with or co-infected with malaria also underscores the shared role of *Anopheles* mosquitoes as vectors for both pathogens. This ecological overlap may contribute to co-transmission and diagnostic ambiguity in endemic regions. Coinfections are common in this setting, and the detection of multiple pathogens in febrile patients underscores the complexity of clinical diagnosis and the need for integrated surveillance strategies. These strategies should account for overlapping transmission ecologies and support the development of pathogen-specific diagnostics to inform appropriate clinical management and public health interventions. ONNV will be managed as malaria with antimalarial medication since ONNV is a self-limiting cause of febrile illness. Syndromic management will suffice for other symptoms. It is important to note that the coinfections reported reflect detection events rather than confirmed concurrent infections. While fever was a common symptom among all patients with detected ONNV, other symptoms varied. There is a need for expanded diagnostic capacity and confirmatory testing in future studies to better understand the clinical relevance of such co-detections.

These findings reinforce the importance of molecular testing in resource-limited settings to accurately identify and monitor emerging or re-emerging arboviral threats. Given that six of seven patients recovered without complications, supportive treatment remains appropriate, though there is a pressing need for broader epidemiologic studies to clarify the burden of ONNV in West Africa.

## CONCLUSION

Clinicians should consider ONNV as a differential diagnosis in malaria-negative febrile patients. Improved diagnostic access and clinical awareness are vital to understanding the true epidemiology of ONNV in Nigeria.
